# Microbiome analysis of Cystic Fibrosis sputum presents higher sensitivity than the conventional bacterial culture

**DOI:** 10.1016/j.bjid.2026.105876

**Published:** 2026-07-02

**Authors:** Fabiana Caroline Zempulski Volpato, Otávio von Ameln Lovison, Daiana de Lima-Morales, Evelyn Kern Almeida, Pabulo Henrique Rampelotto, Andreza Francisco Martins, Paulo José Cauduro Maróstica, Afonso Luís Barth

**Affiliations:** a Hospital de Clínicas de Porto Alegre, LABRESIS – Laboratório de Pesquisa em Resistência Bacteriana, Porto Alegre, RS, Brazil; b Universidade Federal do Paraná, Departamento de Biociências, Palotina, PR, Brazil; c Universidade Federal do Rio Grande do Sul, Programa de Pós-Graduação em Ciências Farmacêuticas, Porto Alegre, RS, Brazil; d Hospital de Clínicas de Porto Alegre, Bioinformatics Core, Porto Alegre, RS, Brazil; e Universidade Federal do Rio Grande do Sul, Programa de Pós-Graduação em Genética e Biologia Molecular, Porto Alegre, RS, Brazil; f Universidade Federal do Rio Grande do Sul, Programa de Pós-graduação em Saúde da Criança e do Adolescente, Porto Alegre, RS, Brazil

**Keywords:** ASV, Lung microbiome, Microbiological community, Culture-independent technique, Cystic Fibrosis

## Abstract

**Background:**

Microbiological communities in the airway of Cystic Fibrosis (CF) patients may be associated with clinical conditions and bacterial exacerbation. The main aim of this study was to establish the correlation between the airway microbiome and the bacteriological culture. We also correlated the microbiome data with the CFTR mutation, presence/absence of leukocytes and hospitalization status of CF patients.

**Aims:**

To establish the correlation between the airway microbiome and the bacteriological culture. We also correlated the microbiome data with the CFTR mutation, presence/absence of leukocytes and hospitalization status of patients.

**Methods and results:**

Sputum collected for routine bacteriological culture of 27 CF patients was submitted to microbiome sequencing. Library of 16S rRNA was prepared using a V3V4 region. The Amplicon Sequence Variants (ASV) obtained from sequencing were compared according with the CFTR mutation and laboratory parameters. Leukocytes in the sputum were evaluated by a differential slide counting in microscopy. The genus *Staphylococcus* and *Pseudomonas* were detected by microbiome analysis in all sputa while *Staphylococcus aureus* was identified in only 19 (70.4%) and *Pseudomonas aeruginosa* in only 9 (33.3%) sputa by bacteriological culture. In 14 specimens the genus *Burkholderia* (*Burkholderia-Caballeronia-Paraburkholderia*) was detected by microbiome analysis; however, the *Burkholderia cepacia* complex was identified in only 8 sputa by bacteriological culture. Lower alpha diversity was directly correlated to the leukocyte presence and hospitalized patients. There was no significant difference in alpha diversity and CFTR mutations.

**Conclusion:**

The use of NSG resources has become an important tool to improve the knowledge of the microbial profile of a CF patient. Our findings contribute to a better understanding of that the evaluation of the airway microbiome of CF patients plays an important role to better understand the pulmonary microbiota and to anticipate the detection of common CF pathogens.

## Introduction

Cystic Fibrosis (CF) patients suffer from recurrent airway bacterial infections which may lead to respiratory failure.^1^ The bacterial colonization of the lung tissue occurs in the early stages of the disease and evolves during time. The microorganism in the lung of CF patients form biofilms (aggregated of cells) which is a growth model that protect them from the action of antibiotics and from the immune response of the host.^1^ Compared with healthy individuals, CF patients present marked changes in the microbiota structure.^2^ Although the main aspects which trigger the CF pulmonary exacerbation are not totally clear, repeated exacerbations are the main factor that leads to the decreasing lung function.^3^ Whelan and Surette^3^ have associated pulmonary exacerbation with an increase in bacterial load of a specific pathogen and an increase in virulence through polymicrobial interactions. Therefore, the CF sputum must be studied by culture-independent techniques in order to evaluate whether these methodologies may provide data about the microbial communities in the airway that improve the knowledge about the pulmonary exacerbation.

Lungs were considered sterile organs mostly due to the fact that culture-dependent techniques sometimes present negative results.^4^ The emergence of culture-independent techniques, such as microbiome analysis with the sequencing of the 16S rRNA gene, has generated data that question the concept of “sterile lung”. Currently, it is considered that the low respiratory tract may be composed of a diverse microbial community, and this leads to new hypotheses related to the pathogenesis of the disease.^4^

Reports in the literature suggest that the microbiome of healthy individuals is more diverse than the microbiome of patients with some chronic lung diseases.^5^^,^^6^ The airway microbiome of patients with CF is quite complex but a few studies reported that the reduction in the diversity of the bacterial community and the predominance of a specific pathogen is associated with age, use of antibiotics, decreased lung function, adhesion of microorganisms and disease progression.^7^, ^8^, ^9^, ^10^, ^11^

This current study describes the microbiome of the airway of different patients with CF with the main aim to evaluate the correlation between the microbiome of the airway and bacteriological culture. We also correlated the microbiome data with the CFTR mutation, presence/absence of leukocytes and hospitalization status of young CF patients.

## Materials and methods

### Study design

This is a cross sectional study that included 27 patients with age between 5‒ and 18 years. The selection of patients was done by convenience of CF individuals attending “Hospital de Clínicas de Porto Alegre”, a tertiary-care, CF reference center in southern Brazil. All participants had confirmed diagnosis of CF by the identification of the CFTR gene mutation. The individuals were classified in four groups, according to CFTR gene: (1) HodeltaF: homozygous for delta F508 mutation; (2) HedeltaF: heterozygous for delta F508; (3) NohodeltaF: homozygous for other non-delta-F mutations; (4) NohedeltaF: heterozygous for other non-delta F mutations. The information of hospitalization status (inpatient or outpatient) was obtained from the medical records.

### Laboratorial parameters

A total of 27 sputa specimens, one per patient, collected during routine medical care were evaluated. Sputa were cultured onto the following solid media: sheep blood agar, chocolate agar, mannitol salt agar, MacConkey agar, and *Burkholderia cepacia* selective agar. Colonies of bacteria that grew in the media with suggestive morphology of common CF pathogens (*Pseudomonas aeruginosa; Staphylococcus aureus; Burkholderia cepacia* complex (Bcc); among others) were identified by the Matrix-Assisted Laser Desorption Ionization-Time Of Flight Mass Spectrometry (MALDI-TOF MS).

The presence or absence of leukocytes in the sputum was evaluated by a differential slide counting in microscopy (100× objective) to identify the white cells of sputum smears stained with May-Grunwald-Giemsa. The presence or absence of leukocytes was used as a parameter to assess the quality of the sputum samples. This approach was adopted because sputum collection in patients with CF is typically performed on a routine basis, often in the absence of clinical signs of pulmonary exacerbation.

### DNA extraction and library preparation

DNA was extracted from 180 μL of CF sputum samples using QIAamp DNA Mini Kit (QIAgen, Valencia CA), with proteinase K pre-treatment (60-minutes at 56 °C), followed by bead-beating with zirconia/silica beads in a FastPrep-24 5G system (Qbiogene, CA), for 30 s at 6.0 m/s (repeated 3-times). Total nucleic acids were eluted in 60 μL of TE (Tris-EDTA Ph 8.0) buffer.

The microbiome of the sputum of the CF patients was performed by 16S rRNA sequencing. Library was prepared according to “16S Metagenomic Sequencing Library Preparation Illumina” protocol, using V3V4 region (primers F: TCGTCGGCAGCGTCAGATGTGTATAAGAGACAGCCTACGGGNGGCWGCCAG R: GTCTCGTGGGCTCGGAGATGTGTATAAGAGACAGGACTACHVGG GTATCTAA). The resulting libraries were pooled in equimolar amounts and sequenced in Illumina MiSeq (Illumina, San Diego, US). To guarantee the reproducibility of technique a set of three sputa were evaluated in triplicate.

### Sequencing analysis

The bioinformatic analysis was performed using the Bioconductor Workflow for Microbiome Data Analysis.^12^ The reads were quality filtered and truncated at position 240. Paired-end joining, determination of Amplicon Sequence Variants (ASV); removal of chimeric sequences, and taxonomic assignment were performed using the DADA2 R package v1.16 algorithm and the SILVA version 138.1 (March 10, 2021). The statistical analysis was performed using the phyloseq R package v1.36.0. The taxonomic data was transformed into relative abundance to detect the differences. Microbiome alpha diversity was calculated according to Shannon and Simpson index; and the statistical significance was calculated using Kruskal–Wallis test. The alpha diversity of the microbiome was correlated to the hospitalization status (inpatient or outpatient), CFTR mutation and with the presence or absence of leukocytes. Data of microbiome richness was estimated using the Abundance-based Coverage Estimators (ACE) index based on the number of ASV detected in each specimen. Richness was interpreted comparatively across samples rather than as an absolute categorical parameter.

Microbiome analysis of sputum specimens were evaluated in triplicate in order to evaluate the reproducibility of the technique.

### Ethics approval

Evaluation of bacteriological culture of sputum and medical record review were approved by the Ethics Committees from “Hospital de Clínicas de Porto Alegre” (CAAE: 23417319.0.0000.5327).

## Results

All 27 sputa specimens were analyzed, and the microbiome data resulted in a total of 1,290,140 reads which generated 2366 taxa. The comparison of triplicate results from a set of three sputa from the same patient showed that our methodology was reproducible, and that sequencing bias was negligible (data not shown).

**Fig. 1:**
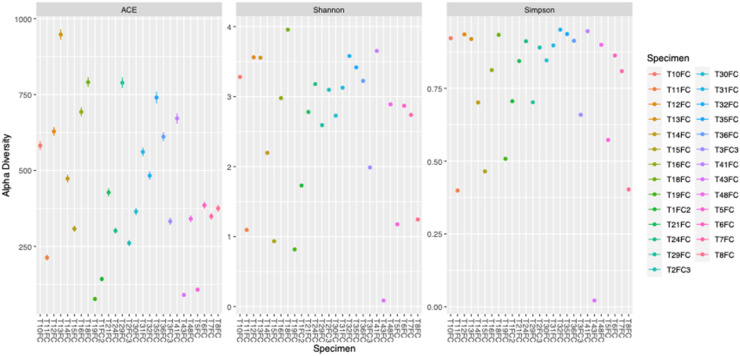
General alpha diversity. Alpha diversity distribution of sputum specimens from CF patients, calculated by Shannon and Simpson index. The richness distribution of sputum specimens from CF patients were calculated by Abundance-Based Coverage Estimators (ACE).

**Fig. 2:**
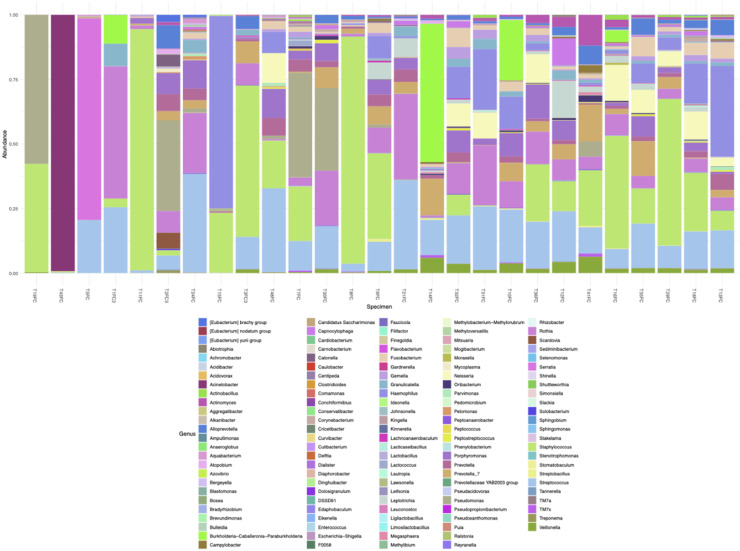
Genus profile of sputum from Cystic Fibrosis patient according to microbiome analysis. Relative abundance of bacterial taxa from 27 different patients with CF according to 16S rRNA. The colored segments represent the relative proportion of ASV in each bar. CF patients were generated in ascending order according to ACE index.

The general alpha diversity was quite variable among different CF patients indicating that there were marked differences in the microbiome composition among the sputa from different patients (Fig. 1). Regarding richness, it was possible to identify five sputa (T1FC2; T5FC; T11FC; T19FC; T43FC) from CF patients which presented a reduced number of ASVs compared with the remaining samples analyzed (Fig. 1 ‒ ACE). In addition, the taxonomic assignments were used to establish the bacterial profile in each specimen, and it was possible to distribute the data in a bar plot of relative abundance according to genus level (Fig. 2). The lower richness of ASVs of the five sputa mentioned above was clearly observed in the bar plot (Fig. 2 – The first five specimens to the left) compared to the other 22 sputa with high richness (Fig. 2). It was also reflected by the predominance of a limited number of bacterial genera in the corresponding bar plot profiles (Fig. 2).

Considering the bacteriological culture, the number of isolates identified in different sputa varied from one to four isolates. It was possible to identify a single isolate in 44.4% (12/27) of the sputa; 33.3% (9/27) of sputa presented two different isolates and 14.8% (4/27) sputa presented three or more different bacterial isolates (Table 1).

**Table 1:** Comparison between the results of the bacteriological culture and microbiome analysis considering the main pathogens of CF (*Staphylococcus, Burkholderia* and *Pseudomonas*) of 27 sputa of CF patients.

**Specimen**	**Bacteriological culture**	**Genus detected by 16S rRNA sequencing (microbiome)**
T1FC2	*Burkholderia cepacia* Complex	*Burkholderia, Staphylococcus* and *Pseudomonas*
T2FC3	*Staphylococcus aureus*	*Burkholderia, Staphylococcus* and *Pseudomonas*
T3FC3	*Pseudomonas aeruginosa* and *Staphylococcus aureus*	*Burkholderia, Staphylococcus* and *Pseudomonas*
T5FC	*Serratia marcescens*	*Staphylococcus* and *Pseudomonas*
T6FC	*Burkholderia cepacia* Complex; *Pseudomonas aeruginos*a and *Staphylococcus aureus*	*Staphylococcus* and *Pseudomonas*
T7FC	*Burkholderia cepacia* Complex; *Pseudomonas aeruginosa* and *Staphylococcus aureus*	*Burkholderia, Staphylococcus* and *Pseudomonas*
T8FC	*Achromobacter* sp and *Staphylococcus aureus*	*Burkholderia, Staphylococcus* and *Pseudomonas*
T10FC	*Burkholderia cepacia* Complex and *Pseudomonas aeruginosa*	*Burkholderia, Staphylococcus* and *Pseudomonas*
T11FC	*Staphylococcus aureus*	*Staphylococcus* and *Pseudomonas*
T12FC	*Staphylococcus aureus*	*Staphylococcus* and *Pseudomonas*
T13FC	*Burkholderia cepacia* Complex and *Staphylococcus aureus*	*Burkholderia, Staphylococcus* and *Pseudomonas*
T14FC	*Burkholderia cepacia* Complex and *Staphylococcus aureus*	*Burkholderia, Staphylococcus* and *Pseudomonas*
T15FC	*Burkholderia cepacia* Complex	*Burkholderia, Staphylococcus* and *Pseudomonas*
T16FC	*Burkholderia cepacia* Complex and *Staphylococcus aureus*	*Burkholderia, Staphylococcus* and *Pseudomonas*
T18FC	*Staphylococcus aureus*	*Staphylococcus* and *Pseudomonas*
T19FC	*Pseudomonas aeruginosa* and *Staphylococcus aureus*	*Burkholderia, Staphylococcus* and *Pseudomonas*
T21FC	Microbiota growth	*Staphylococcus* and *Pseudomonas*
T24FC	Microbiota growth	*Burkholderia, Staphylococcus* and *Pseudomonas*
T29FC	*Staphylococcus aureus*	*Burkholderia, Staphylococcus* and *Pseudomonas*
T30FC	*Pseudomonas aeruginosa*	*Staphylococcus* and *Pseudomonas*
T31FC	*Staphylococcus aureus*	*Staphylococcus* and *Pseudomonas*
T32FC	*Acinetobacter ursingii; Pseudomonas putida; Serratia marcescens and Staphylococcus aureus*	*Staphylococcus* and *Pseudomonas*
T35FC	*Staphylococcus aureus*	*Staphylococcus* and *Pseudomonas*
T36FC	*Pseudomonas aeruginosa* and *Staphylococcus aureus*	*Staphylococcus* and *Pseudomonas*
T41FC	*Pseudomonas aeruginosa and Staphylococcus aureus*	*Staphylococcus* and *Pseudomonas*
T43FC	Acinetobacter *baumannii/calcoaceticus* Complex	*Staphylococcus and Pseudomonas*
T48FC	*Pseudomonas aeruginosa* and *Staphylococcus aureus*	*Burkholderia, Staphylococcus* and *Pseudomonas*

The pathogens (genus *Staphylococcus, Burkholderia* and *Pseudomonas*) commonly associated with the disease in the airway of CF were frequently detected in the microbiome of the sputa of the CF patients but varied in prevalence and relative abundance. The genus *Staphylococcus* and *Pseudomonas* were detected by microbiome analysis in all 27 sputa but *Staphylococcus aureus* was identified in only 19 (70.4%) and *Pseudomonas aeruginosa* in only 9 (33.3%) sputa by bacteriological culture. The genus *Burkholderia* (*Burkholderia-Caballeronia-Paraburkholderia*) was detected by microbiome analysis in 14 sputa and the *Burkholderia cepacia* complex was identified in 8 sputa by bacteriological culture. However, the genus *Burkholderia* was not detected in the microbiome of one sputum (sample T6FC) which presented positive result for Bcc in the bacteriological culture (Table 1).

**Fig. 3:**
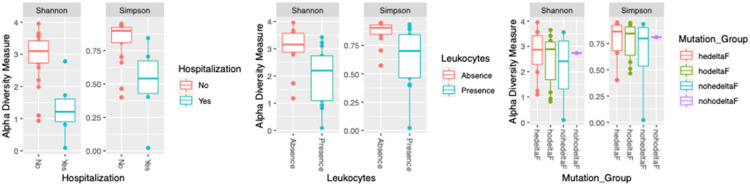
Correlation of the Alpha diversity with hospitalization, presence of leukocyte and CFTR group mutation. Alpha diversity of sputum specimens from CF patients by boxplots representing Shannon and Simpson equations were plotted in relation to hospitalization status (inpatient or outpatient), presence or absence of leukocyte and CFTR group mutation. The patients were classified in four groups, according to CFTR gene: 1) HodeltaF: homozygous for delta F508 mutation; 2) HedeltaF: heterozygous for delta F508; (3) NohodeltaF: homozygous for other non-delta F mutations; 4) NohedeltaF: heterozygous for others non-delta F mutations.

A total of 22.2% (6/27) of sputum specimens were obtained from hospitalized patients and 81.5% (22/27) were from outpatients. In our study, the alpha diversity has demonstrated that lower diversity was significantly associated with hospitalization (Fig. 3A; Shannon: *p* = 0.003545 and Simpson: *p* = 0.007301). Moreover, less diverse bacterial communities were also associated with the presence of leukocytes in the sputum (Fig. 3B; Shannon and Simpson: *p* = 0.005675) in our study.

Eleven patients were homozygous for delta F508 mutation; 11 were heterozygous for delta F508; only one patient was homozygous for other CFTR mutations; and 4 were heterozygous for other CFTR mutations. There was no significant difference in alpha diversity and the groups of mutations (Fig. 3C; Shannon: *p* = 0.9652 and Simpson *p* = 0.9872).

## Discussion

For decades, cultured-dependent methods have provided evidences that the colonization/infection in CF airways were mostly due to a few bacterial species. Therefore, the treatment of the airway infections and pulmonary exacerbations in CF were usually based in the results of the bacteriological culture of sputum of CF patients.^13^^,^^14^ In fact, the sputum specimens from CF patients were considered as representative of lower airway despite the fact that this sample transit through the upper respiratory system during collection. It has been considered that the high viscosity, characteristic of the CF sputum, minimizes its mixing with upper respiratory tract during collection.^15^

The emergence of the molecular techniques has shed light on the complex structure of the lung microbiota. Data generated by metagenomics indicate that bacterial community structure and composition may represent an important factor in defining the role of the microbiome in host health status.^8^ We evaluated the ASVs as the parameter of the taxonomic assignment and it was possible to identify a different range of alpha diversity in 27 sputum specimens, indicating a considerable difference among microbiome among patients. Therefore, it is possible to indicate that the pulmonary microbiota in CF is particular to each patient as has already been demonstrated by others studies.^3^^,^^9^^,^^16^ This study is one of the first studies to describe the lung microbiome of CF patients analyzed by ASV.

Noteworthy, it was possible to observe that the analysis of the microbiome from CF patients was able to identify the genus of the common pathogens (*Pseudomonas, Staphylococcus* and *Burkholderia*) usually identified by the bacteriological culture. It has to be considered that different pathogens seem to be age specific in CF as *Staphylococcus aureus* which is considered the most prevalent in the early life and *Pseudomonas aeruginosa* and *Burkholderia cepacia* complex being the pathogens more often found in the adulthood.^13^ In this study, the genus *Staphylococcus* was detected in all microbiomes of sputa and this is in line with the high prevalence of *Staphylococcus aureus* in the airway specimens from young CF patients although this bacterium tends to persists as chronic colonizers in the bronchial tree of CF.^15^ Infection by *S. aureus* in CF is usually associated with nasal carriage of *S. aureus* to the lower airway in the young CF patient.^17^ In fact, modification of the mucus composition in CF usually leads to the airway microenvironment changes causing dysbiosis which ultimately prompt to an increase of colonization by *S. aureus*.^13^

The disease in the CF lung is associated with a chronic non-resolving inflammation and recurrent bacterial colonization/infection of the airways. It is estimated that 80% to 95% of patients with CF will succumb to respiratory failure caused by chronic bacterial infection and concomitant airway inflammation.^1^ Lungs of young CF patients are often colonized by microorganisms such as *Staphylococcus aureus* and *Haemophilus influenzae*, which can damage the epithelial surfaces of the airway allowing the adhesion of other bacteria, in particular, *Pseudomonas aeruginosa.*^1^ In this study we demonstrated the presence of the genus *Pseudomonas* in all sputa from CF young patients analyzed by microbiome and this is an important finding as *P. aeruginosa* is the main bacteria associated to decline of the lung function in CF.^1^ It is important to consider that the presence of 16S sequencing of *Pseudomonas* spp as detected by microbiome analysis cannot be directly related to the presence of viable bacteria in the CF airway. However, considering that the antibiotic treatment for *P. aeruginosa* in CF is more effective as sooner as the bacteria is detected in the bacteriological culture, a methodology with high sensitivity, such as microbiome analysis, would anticipate the presence of *Pseudomonas* spp. and favor an even early treatment. Leite et al.^16^ suggested that metagenomics information may contribute to improve the treatment of CF patients in particular for patients with negative results for airway pathogens in the conventional bacteriological culture. These authors described the case of one CF patient whose clinical status was worsening but the bacteriological culture did not present any important information. However, the microbiome analysis of the sputum of the patient indicated an increase in the abundance of *Pseudomonas* spp and this information was crucial for the decision of the clinicians to change the antimicrobial treatment with an important clinical improvement of the patient.^16^

In our study, the microbiome analysis detected the ASV of *Burkholderia-Caballeronia-Paraburkholderia* in 14 of 27 sputa whereas the *Burkholderia cepacia* complex was observed in only 8 sputa by bacteriological culture. *Burkholderia cepacia* complex is a pathogen often associated with reduced survival and increased risk to death due to the “cepacia syndrome”.^18^ *Burkholderia* is a bacterial genus that contains a large number of species, with the current count being around 100.^19^ Therefore, as mentioned above with *P. aeruginosa*, it may be important to use data of microbiome analysis to consider the presence of *Burkholderia cepacia* complex in the sputum of CF.

Noteworthy, an eventual decrease in diversity of the microbiome is usually associated with an increase in the dominance of a specific taxon^8^ and this appears to be the case of the specimen T43FC in our study (Fig. 2). The specimen T43FC presented a high abundance of the genus *Acinetobacter* and in the conventional bacteriological culture only *Acinetobacter baumannii/calcoaceticus* complex was identified. This is not a pathogen commonly associated with the pulmonary infection in patients with CF, however this specific patient was submitted to lung transplantation ten days before the sputum was collected for this study. Although the *Acinetobacter baumannii/calcoaceticus* complex is not normally associated with infection in CF patients, the identification of this pathogen in high prevalence in both methods (microbiome and culture) indicates that this bacterium was probably causing infection in this patient. Therefore, data corroborated by both microbiome and culture may also be important to warrant the best clinical treatment of CF patients.

Furthermore, our study provided information on the relationship between airway microbiota, hospitalization status, and the presence or absence of leukocytes in the sputum specimens. Our results demonstrated that the inpatients and the presence of leukocytes in sputum were significantly associated with a lower alpha diversity. Therefore, it is possible to suggest a correlation between lower diversity and the infection process in the airway of CF patients as the polymorphonuclear leukocytes represent the first cells migrating into the pulmonary tissue to combat infection.^20^ Metagenomic analysis indicated that alpha diversity was significantly lower in hospitalized patients (*p* < 0.05), which is consistent with the suggestion that increased microbiome diversity may be associated with a better prognosis in patients with Cystic Fibrosis. An important limitation of this study is the heterogeneity of the patient population and the relatively small sample size, which may have limited more in-depth analyses and restrict definitive conclusions regarding prognostic implications.

Reduced microbial diversity and dysbiosis have been consistently associated with disease severity in CF, although their role as prognostic or therapeutic targets remains to be fully established.^21^ Although the alpha diversity was not significantly different for each group of CFTR mutations, it was possible to observe a lower diversity for nohedeltaF group. A trend cannot be excluded, as this group was composed of the sputum of the patient T43FC which presented the lowest diversity observed among the sputa analyzed. A report in literature suggests that mutations that turn the CFTR non-functional are associated with a decrease of the airway bacterial diversity.^8^ Despite this, it is not yet clear whether this is directly related to the severity of the mutation or due to the antimicrobial treatment required to manage these patients.^8^ Recent studies suggest that the organization and structure of the airway microbiome, rather than only its taxonomic composition, may be associated with different patterns of pulmonary exacerbations and potentially influence treatment response in patients with Cystic Fibrosis.^22^

Although chronic airway infection in CF is recognized as polymicrobial, clinical surveillance and treatment strategies remain primarily guided by conventional culture-based methods.^23^ The use of the next-generation sequencing resources has become an important tool to improve the knowledge of the microbial profile of a CF patient. We understand that the evaluation of the airway microbial community, considering the diversity and the abundance of pathogens, may provide important information is an important information to be used as an aid to improve the clinical management of CF patients. We found that the microbiome analysis is more sensitive than the conventional bacterial culture and this may potencially be useful to anticipate/change the antibiotic treatment of CF patients. However, further studies are needed to validate these findings and to determine their impact on clinical outcomes.

## Authors' contributions

FCZV, DLM and ALB conceived and established the design of the study; FCZV, DLM and EKA were responsible for DNA extraction and the Next Generation Sequencing; FCZV, OAL, PHR and AFM performed the bioinformatic analysis; PJCM was responsible for the evaluation of the clinical data; FCZV wrote the manuscript; ALB, PJCM and AFM revised the writing. All the authors approved the final version of the manuscript.

## Patient and public involvement

Patients and/or the public were not involved in design, or conduct, or reporting, or dissemination plans of this research.

## Patient consent for publication

Not applicable.

## Funding

This work has been funded by INPRA ‒ Instituto Nacional de Pesquisa em Resistência Antimicrobiana ‒ Brazil (INCT/CNPq: 465718/2014-0 and INCT/FAPERGS: 17/2551-0000514-7) and by Fundo de Financiamento e Incentivo à Pesquisa (Fipe/HCPA) (Project no.° 2019-0659).

## Data availability

The datasets generated during and/or analyzed during the current study are available from the corresponding author on reasonable request.

## Conflicts of interest

The authors declare no conflicts of interest.
